# A comparison of comorbid headache between patients with temporal lobe epilepsy and juvenile myoclonic epilepsy

**DOI:** 10.1038/s41598-023-43705-7

**Published:** 2023-10-08

**Authors:** Shujiang Zhang, Jinmei Li, Dong Zhou

**Affiliations:** 1 Department of Neurology, West China Hospital of Sichuan University, Chengdu, China; 2 Department of Neurology, The Affiliated Hospital of Southwest Medical University, Luzhou, China

**Keywords:** Diseases, Neurology

## Abstract

Headache is one of the most common symptoms of epilepsy comorbidities. However, the relationship between the epilepsy and headache still needs clarification. Previous studies mostly investigated the overall incidence and clinical features of the headache in patients with the epilepsy. Temporal lobe epilepsy (TLE) and juvenile myoclonic epilepsy (JME) are the common types of focal epilepsy and generalized epilepsy, respectively. Nevertheless, there was no study comparing the clinical features of headache between TLE and JME. This study aimed to analyze the headache features of these two types of epilepsy. Patients with either TLE or JME diagnosed with headache and referred to the West China Hospital of Sichuan University were consecutively recruited from June 2021 to June 2022. The duration of epilepsy was longer than 6 months in these patients. Data on headache and epilepsy were obtained through face-to-face questionnaires. The headache was classified according to the International Classification Headache Disorders-3rd edition (ICHD-III) criteria. χ^2^-test, t-test, rank-sum test, logistic regression modeling and Mann Whitney test were used to compare the clinical differences of the headache in TLE and JME. A total of 151 TLE patients and 30 JME patients were enrolled in this study. There was no significant difference in the family history of headache, epilepsy durations, headache types, proportion receiving analgesic therapy, the frequency of inter-ictal headache (inter-IH), and the quality of life in epilepsy -10 inventory (QOLIE-10) between the TLE and JME patients. Patients in the TLE group were significantly older (p = 0.004), and a lower percentage of them had a family history of epilepsy (p = 0.007) compared with the JME patients. The proportion of cases with refractory epilepsy was higher in the TLE group than that in the JME group (p < 0.001). The types of seizures in the TLE group varied from those in the JME group (p < 0.001). The composition of the antiseizure medications (ASM) applied in the TLE group differed from that in the JME group (p = 0.047), and the usage of oxcarbazepine was more frequently in the TLE group than in the JME group (p = 0.003). There was no difference in the headache types among patients with TLE or JME. Specifically, 67 (44.37%), 12 (7.95%), and 118 (7.95%) patients were found with inter-IH, pre-ictal headache (Pre-IH) and post-ictal headache (Post-IH) in the TLE group; while 8 (26.67%), 4 (13.33%) and 26 (86.67%) patients had inter-IH, Pre-IH and Post-IH in the JME group. Thirty-nine patients in the TLE group and 4 patients in the JME group were identified with more than one type of headaches, respectively. Tension-type headache (TTH) were found in 38 patients (25.17%) in the TLE group and 3 patients (10.00%) in the JME group, respectively; migraines were found in 10 patients (6.62%) in the TLE group and in 2 patients (6.67%) in the JME group. Patients in the TLE group had a higher headache-attributed lost time-90 days (HLT-90) score than those in the JME group (p = 0.019). The proportion of patients with inter-IH accompanied by nausea in the TLE group was higher than that in the JME group (p = 0.029), while the proportion of patients with frontal headache was lower than that in the JME group (p < 0.05). There was no significant difference in headache severity, quality, headache nature, unilateral/bilateral, and headache duration either in inter-IH or peri-ictal headache (Peri-IH) between the two groups. The logistic regression analysis suggested that except for HLT-90 (AUC = 0.622, p = 0.027), other factors were not found to be correlated with refractory epilepsy. The clinical features of headache differed between TLE and JME patients. TLE patients had a higher ratio of refractory epilepsy, more headache time loss compared with JME patients. HLT-90 was associated with the occurrence of refractory epilepsy in TLE patients. Taken together, we suggested that the comorbid headache may essentially be different between TLE and JME patients.

## Introduction

Headache is one of the common comorbid symptoms in patients with epilepsy. The prevalence of headache in unselected epilepsy patients was reported to be 52–66%^1–3^. The relationship between the comorbid headache and different types of epilepsies is still not fully understood^4^. Previous literature has shown that patients with headache comorbidity have lower quality of life than patients who didn’t. According to the temporal profile of epilepsy, the headache in epilepsy can be divided into inter-ictal headache (inter-IH) and peri-ictal headache (peri-IH). The headache occurred between seizures is known as inter-IH^5^. Peri-IH, known to be correlated with seizure, includes pre-ictal headache (pre-IH), ictal headache (IH) and post-ictal headache (post-IH). Studies have reported that generalized tonic–clonic seizures (GTCS) appeared to be more frequently accompanied by post-IH than the other types of seizure^6–8^. The diagnosis and treatment of comorbid headache are insufficient among epilepsy patients, especially the post-IH.

Literature has recorded that the inter-IH frequency in patients with epilepsy ranged from 19 to 34.3%, and the incidence of peri-IH ranged from 40 to 60%^9,10^. Different headache types in epilepsy patients may involve different pathophysiology. Of all types of headaches, migraine is considered most closely related to epilepsy, and there may be some common underlying pathophysiological mechanisms shared by migraine and epilepsy^11^. It’s noted that they have some common risk factors and triggering factors, and that the therapeutic drugs are similar, such as valproic acid and topiramate. Cortical spreading depression is a relatively well recognized mechanism for the occurrence of epilepsy comorbid migraine^12^, while post-IH may be related to post-attack cerebral vasodilatation^13^, and some special epilepsy may also involve genetic background, such as SCN1A, which has been linked to epilepsy and migraine^14^. The migraine frequency ranged from 8 to 24% in epilepsy patients according to previous studies^15,16^. Temporal lobe epilepsy (TLE) is one of the common types of focal epilepsy and is also among the most common types of refractory epilepsy. Juvenile myoclonic epilepsy (JME) is a type of idiopathic generalized epilepsies characterized by myoclonic jerks, GTCS, and absence seizures^17^. TLE and JME are totally different in terms of seizure types, selection of antiepileptic drugs, as well as prognosis. Overall, JME has a better response to antiepileptic drugs than TLE. There are few previous studies on the relationship between epilepsy subtypes and headache, and the relationship between classification of epilepsy and headache is unclear. Whether the comorbid headache in TLE varies from that in JME is still unknown. We here aimed to determine the clinical characteristics of the headache of TLE and JME patients. We also explored the correlation between the clinical factors of headache and the occurrence of refractory epilepsy among TLE patients, in order to discover the possible headache risk factors for refractory epilepsy.

## Methods

The study was approved by the institutional review board of West China Hospital of Sichuan University (2022301). All participants provided detailed written information on the study and written informed consent was obtained in accordance with the Declaration of Helsinki.

### Study design and participants

This is a cross-sectional study enrolling both in-patients and out-patients at the Epilepsy Center of West China Hospital of Sichuan University from June 2021 to June 2022. The inclusion criteria include an age ≥ 18 years and the presence of headache symptoms in TLE and JME patients. The duration of epilepsy was longer than 6 months in these patients. Interrogation interviews were conducted to determine whether the patient had headaches during the course of epilepsy. Once the patient had confirmed the occurrence of headache, a face-to-face questionnaire survey would be performed for characterizing the headache types, in other words, whether it was an inter-IH or peri-IH. Two neurologists were responsible for classifying the clinical characteristics of headache according to the ICHD-III diagnostic criteria^18^, and epileptic seizures is classified according to the 2017 International League Against Epilepsy (ILAE) Commission report^19^. Patients with a duration of epilepsy less than 6 months, a single seizure, or a severe intellectual disability, and those who cannot cooperated with the questionnaire were excluded.

### Data collection

**Figure 1:**
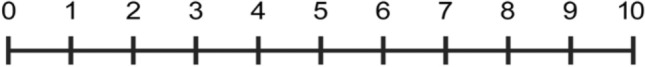
Visual analog scale (VAS). Mild pain: 1–3; Moderate pain: 4–6, severe pain: > 7.

Patient data were obtained by field investigation; demographic data were collected for all patients. The information collected on epilepsy includes the age at the onset of epilepsy, disease duration, seizure types, the frequency of seizures, the family history of epilepsy, whether there existed head traumas or febrile convulsions, the current antiepileptic medications, and the quality of life in epilepsy -10 inventory (QOLIE-10). The characteristics of headache include family history of headache, duration of headache, headache types, headache sites, quality of pain, headache severity according to the visual analog scale (VAS), headache quality, lateralization, frequency of inter-IH, usage of analgesic therapy. Headache severity was assessed based on the patient's memory of previous feeling of pain and indicated corresponding number on VAS (Fig. 1). Concomitant symptoms were also collected, such as nausea, emesis, photophobia, phonophobia, and whether symptoms worsening with physical activity. For those who were diagnosed with migraine, whether aura, like photophobia, flash, scotoma, blurred vision, visual field defects, sensory and language disorders, occurred before headache onset was collected.

For peri-IH patients, the interview focused on questions on timing of onset (pre-ictal, ictal or post-ictal headaches) and frequency of headache. The frequency was classified into the following categories: always, occurring after every seizure; often, occurring after more than two-thirds of seizures; sometimes, occurring after less than half of seizures; and rarely, occurring only for a few times. We also calculated the headache-attributed lost time-90 days (HLT-90). Finally, two neurologists respectively specialized in headache and epilepsy reviewed the clinical data of all the patients again to ensure the accurate classification.

### Definitions and classifications

Intractable epilepsy is defined as persistent seizures despite a trial of at least 2 appropriately chosen ASM at optimal doses. Inter-IH manifested the headache during the interval period of epilepsy and it was considered not related to the epileptic seizure. Pre-IH develops within 24 h before the seizure^10,20,21^. Ictal headache develops during the seizure^10^. Post-IH usually develops within 3 h following a partial or generalized seizure and often resolves within 72 h after the seizure^18^. Inter-IH was divided into migraine (with or without aura), tension-type headache (TTH), and other primary headaches according to ICHD-III criteria^18^. All patients completed brain MRI, secondary headaches in structural epilepsies were not included in our study.

### Statistical analysis

Clinical variables in the TLE group and JME group were analyzed. We performed χ2 -test to compare the categorical variables. T-test and Mann Whitney U test were performed to evaluate the continuous variables with a symmetrical or asymmetrical distribution, respectively. A logistic regression model was used to calculate odds ratio (OR) and 95% confidence interval (CI) to assess the association between dependent and independent variables. Statistical significance was set at p value < 0.05. Statistical analyses in this study were performed with SPSS25.0.

### Ethics approval and consent to participate

The Ethics Committee of West China Hospital of Sichuan University reviewed and approved the study. Informed consent was obtained for all participants.

## Results

**Figure 2:**
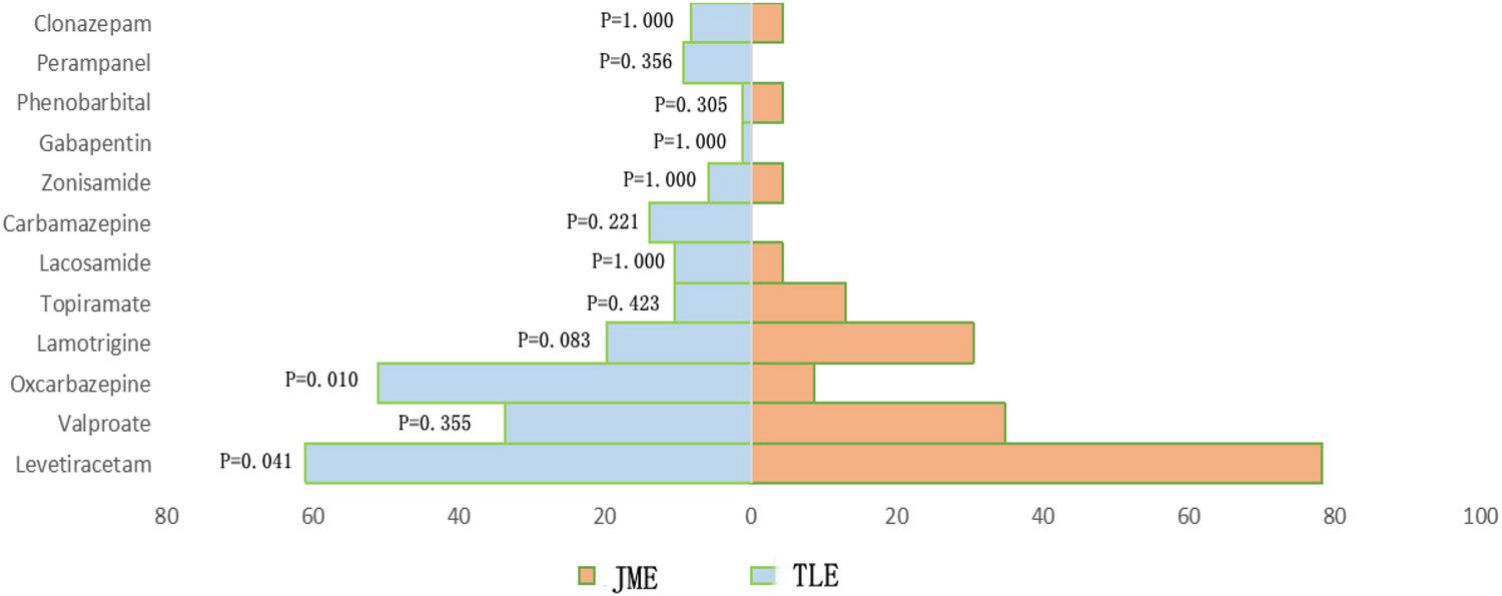
Details of the use of ASM in the TLE and JME groups. Chi-square test or Fisher exact test was used for statistical analysis. *ASM* antiseizure medications, *TLE* temporal lobe epilepsy, *JME* juvenile myoclonic epilepsy.

A total of 151 TLE cases and 30 JME cases were enrolled. The main clinical features of the patients are listed in Table 1, and the clinical features of epilepsy-related headaches are displayed in Table 2. There were 88 females in the TLE group and 18 females in the JME group. The mean age of the TLE patients and the IME patients at the interview was 30.65 ± 9.70 years and 24.57 ± 3.46 years, respectively. In this study, TLE patients were significantly older than JME patients (p = 0.004). The mean age at epilepsy onset in the TLE and JME patients was 18.15 ± 11.00 years and 14.62 ± 5.80 years, respectively. The patients' mean epilepsy duration in TLE group was 12.44 ± 9.61 years; the patients' mean epilepsy duration in JME group was 9.97 ± 5.71 years. Patients in the JME group more frequently had a family history of epilepsy than patients in the TLE group (p = 0.007), and the incidence of intractable epilepsy in the TLE group was higher than that in the JME group (p < 0.001). The types of seizures differed in the two groups (p < 0.001). Specifically, 21 patients (13.91%) developed complex partial seizure (CPS), 33 patients (21.85%) developed GTCS, and 97 patients (64.24%) developed both CPS and GTCS in the TLE group; while in the JME group, 20 patients (66.67%) developed GTCS, 1 patient (3.33%) developed absence, and 9 patients (30.00%) developed absence + GTCS. Twenty-two patients (14.57%) had seizures yearly or in a less frequency, 84 patients (55.63%) had monthly seizures, 29 patients (19.21%) had weekly seizures, and 16 patients (10.60%) had daily seizures in the TLE group. Twelve patients (40.00%) had seizures yearly or in a less frequency, 17 patients (56.67%) had monthly seizures, and 1 patient (3.33%) had monthly seizures in the JME group. In the TLE group, 37 patients (24.50%) were treated with antiseizure medications (ASM) monotherapy, 100 patients (66.23%) received polytherapy (≥ 2 ASM), and 14 patients (9.27%) had not received any treatment (Fig. 2); whereas in the JME group, 9 patients (30.00%) received ASM monotherapy, 20 patients (66.67%) received polytherapy, and only 1 patient (3.33%) had no therapy. In terms of ASM use, the proportion of the oxcarbazepine usage in the TLE group was higher than that in the JME group (p = 0.010), and the levetiracetam usage in the TLE group was lower than that in the JME group (p = 0.041).

**Table 1:** Clinical features of TLE and JME patients.

Group	TLE	JME	p
Total number	151	30	–
Femal/male^@^	88/63	18/12	1.000
Age (years, mean ± SD)^#^	30.65 ± 9.70	24.57 ± 3.46	**0.004**
Age at epilepsy onset (years, mean ± SD)^#^	18.15 ± 11.00	14.62 ± 5.80	0.217
Duration of epilepsy (years, mean ± SD)^#^	12.44 ± 9.61	9.97 ± 5.71	0.563
Family history of epilepsy^@^	4 (2.65)	5 (16.67)	**0.007**
Intractable epilepsy^@^	95 (62.91)	1 (3.33)	** < 0.001**
Seizure type^@^			
CPS^$^	21 (13.91)	0	** < 0.001**
GTCS^$^	33 (21.85)	20 (66.67)	
Absence	0	1 (3.33)	
CPS and GTCS^$^	97 (64.24)	0	
Absence and GTCS^$^	0	9 (30.00)	
Frequency of seizures^@^			
Daily	16 (10.60)	0	**0.001**
Weekly	29 (19.21)	1 (3.33)	
Monthly	84 (55.63)	17 (56.67)	
Yearly or less	22 (14.57)	12 (40.00)	
ASM use^@^			
Monotherapy	37 (24.50)	9 (30.00)	0.591
Polytherapy	100 (66.23)	20 (66.67)	
No therapy	14 (9.27)	1 (3.33)	
QOLIE-10 (median)^#^	25.5	24	0.307
Family history of headache^@^	10 (6.62)	1 (3.33)	0.694
Headache types^@^			
Inter-IH	67 (44.37)	8 (26.67)	0.211
Pre-IH	12 (7.95)	4 (13.33)	
Post-IH	118 (78.15)	26 (86.67)	
Inter-IH^@^			
Migraine*	10 (6.62)	2 (25.00)	0.471
TTH	38 (25.17)	3 (37.50)	
Unclassified	19 (12.58)	3 (37.50)	
Analgesic therapy^@^	27 (17.88)	5 (16.67)	1.000
HLT-90 (day, mean ± SD) ^#^	8.90 ± 19.49	1.22 ± 2.15	**0.019**
Etiology^@^			
Infection	23	–	–
Unknown	128	–	–

Figures are given as N (%), unless otherwise stated.

The numbers in bold indicate statistical significance between two groups.

*TLE* temporal lobe epilepsy, *JME* juvenile myoclonic epilepsy, *SD* standard deviation, *CPS* complex partial seizure, *GTCS* generalized tonic clonic seizure, *ASM* antiseizure medications, *QOLIE-10* quality of life in epilepsy-10 inventory; Inter-IH, inter-ictal headache, *Pre-IH* pre-ictal headache, *Post-IH* post-ictal headache, *TTH* tensiontype headache, *HLT-90* headache-attributed lost time-90 days.

^#^T-test and Mann Whitney U test were performed to evaluate continuous variables with a symmetrical or asymmetrical distribution respectively.

^@^Fisher-Freeman-Halton extension of the Fisher exact test was used for count data.

^$^p < 0.05.

*Only three patients with migraine in the TLE group had aura.

**Table 2:** Clinical features of epilepsy-related headaches.

Group	Inter-IH			Pre-IH			Post-IH		
	TLE	JME	P	TLE	JME	P	TLE	JME	P
	N = 67	N = 8		N = 12	N = 4		N = 118	N = 26	
Severity*^@^									
Mild	22 (32.84)	5 (62.50)	> 0.05^&^	3 (25.00)	1 (25.00)	> 0.05***	12 (10.17)	3 (11.54)	> 0.05^▲^
Moderate	44 (65.67)	3 (37.50)		6 (50.00)	3 (75.00)		77 (65.25)	21 (80.77)	
Severe	1 (1.49)	0		3 (25.00)	0		29 (24.57)	2 (7.69)	
Accompanying symptoms^@^									
Nausea	10 (14.93)	2 (25.00)	0.606	4 (33.33)	1 (25.00)	1.000	30 (25.42)	7 (26.92)	1.000
Emesis	1 (1.49)	2 (25.00)	**0.029**	1 (8.33)	0	1.000	15 (12.71)	0	0.073
Photophobia	10 (14.93)	2 (25.00)	0.606	1 (8.33)	0	1.000	8 (6.78)	0	0.351
Phonophobia	10 (14.93)	3 (37.50)	0.136	3 (25.00)	0	1.000	10 (8.47)	0	0.209
Aggravation by moderate physical activity^@^	18 (26.87)	2 (25.00)	1.000	7 (58.33)	1 (25.00)	0.569	54 (45.76)	7 (26.92)	0.085
Quality									
Pulsating	5 (7.46)	2 (25.00)	0.139	1 (8.33)	0	1.000	7 (5.93)	1 (3.85)	0.908
Pressing/tightening^@^	34 (50.74)	2 (25.00)		7 (58.33	3 (75.00)		77 (65.25)	16 (61.54)	
Other	21 (31.34)	4 (50.00)		3 (25.00)	1 (25.00)		21 (17.80)	5 (19.23)	
Uncertain	7 (10.45)	0		1 (8.33)	0		13 (11.02)	4 (15.38)	
Lateralization									
Unilateral	22 (32.84)	3 (37.50)	1.000	4 (33.33)	1 (25.00)	1.000	27 (22.88)	4 (15.38)	0.598
Bilateral	45 (67.16)	5 (62.50)		8 (66.67)	3 (75.00)		91 (77.12)	22 (84.62)	
Location determined^※^^@^									
Frontal	4 (5.97)	3 (37.50)^$^	**0.009**	1 (8.33)	0	0.447	4 (3.39)	1 (3.85)	**0.046**
Temporo	38 (56.72)	3 (37.50)		7 (58.33)	3 (75.00)		66 (55.93)	10 (38.46)	
Occipital	7 (10.45)	0		1 (11.11)	0		14 (11.86)	1 (3.85)	
Parietal	19 (28.36)	0		3 (25.00)	0		22 (18.64)	6 (23.08)	
Full head	4 (5.97)	0		1 (8.33)	0		22 (18.64)	4 (15.38)	
Uncertain	3 (4.48)	3 (37.50)^$^		0	1 (25.00)		2 (2.33)	4 (15.38)^$^	
Median duration of headache (h)^#^	3	0.5	0.203	1.0	0.08	0.781	5.00	3.00	0.566
Frequency^⁜@^	–	–							
Always	–	–		3 (25.00)	1 (25.00)	1.000	92 (77.97)	22 (84.62)	0.676
Often	–	–		4 (33.33)	2 (50.00)		10 (8.47)	3 (11.54)	
Sometimes	–	–		4 (33.33)	1 (25.00)		12 (10.17)	1 (3.85)	
Rare	–	–		1 (8.33)	0		4 (3.39)	0	

Figures are given as N (%), unless otherwise stated.

The numbers in bold indicate statistical significance between two groups.

*Inter-IH* inter-ictal headache, *Pre-IH* pre-ictal headache, *Post-IH* post-ictal headache, *TLE* temporal lobe epilepsy, *JME* juvenile myoclonic epilepsy.

^#^T-test and Mann Whitney U test were performed to evaluate continuous variables with a symmetrical or asymmetrical distribution respectively.

^@^Fisher-Freeman-Halton extension of the Fisher exact test was used for count data.

*Headache severity was measured by visual analog scale (VAS).

^&^We conducted a two-way analysis of variance (ANOVA) on the types of seizures, types of drugs taken and the frequency of GTCS episodes, the specific p-values were 0.542, 0.330, 0.464 respectively.

***We conducted ANOVA on the types of seizures, types of drugs taken and the frequency of GTCS episodes, the specific p-values were 0.367, 0.269, 0.462 respectively.

^▲^We conducted ANOVA on the types of seizures, types of drugs taken and the frequency of GTCS episodes, the specific p-values were 0.662, 0.183, 0.312 respectively.

^※^Some of the patients had headaches located in more than one site.

^$^p < 0.05.

^⁜^Frequency was classified as: always, occurring after every seizure; often, occurring after more than two-thirds of seizures; sometimes, occurring after less than half of seizures; and rarely, occurring for only a few times.

**Figure 3:**
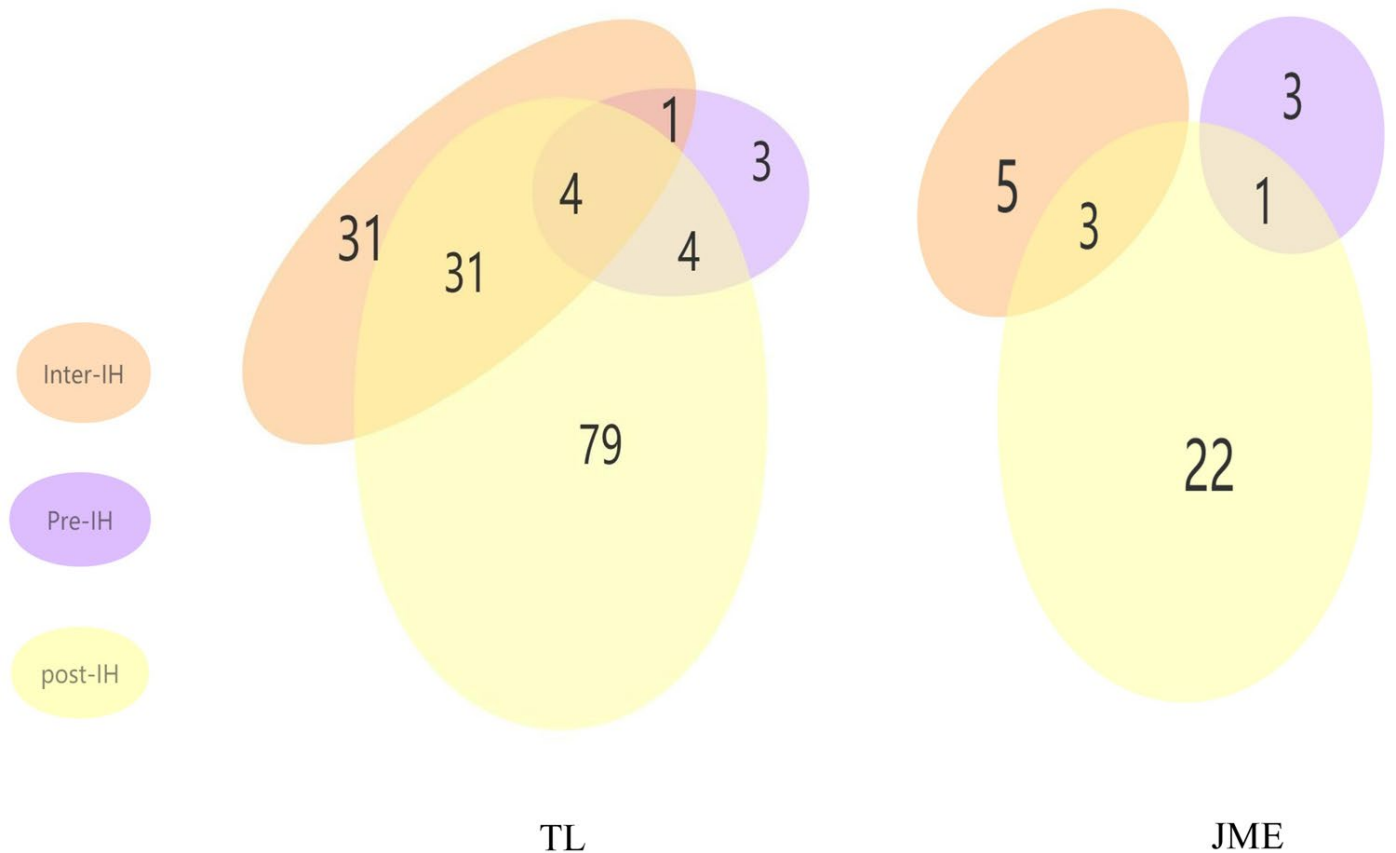
Venn diagram of headache types in the TLE and JME groups. *TLE* temporal lobe epilepsy, *JME* juvenile myoclonic epilepsy.

**Figure 4:**
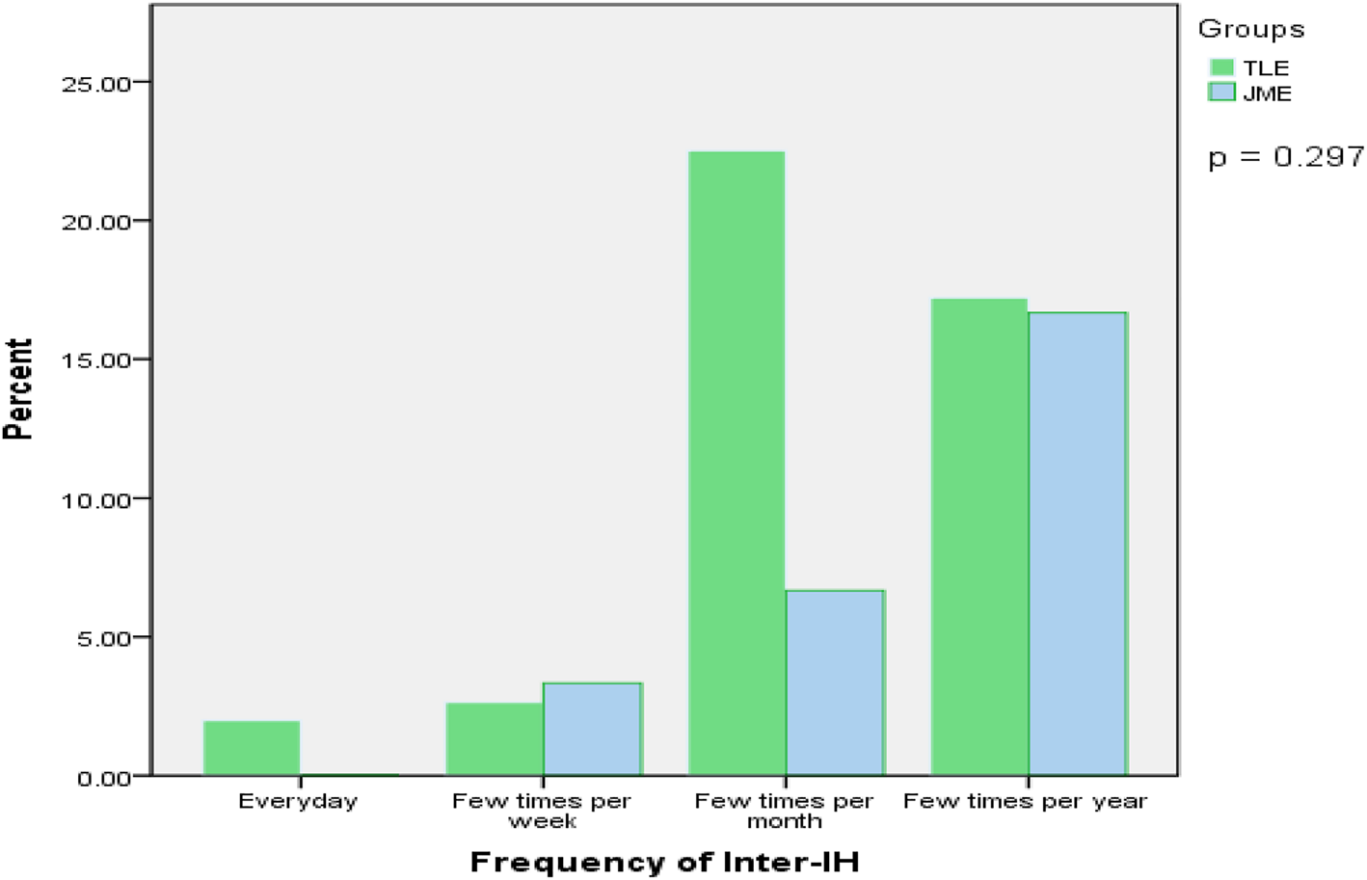
Details of inter-IH frequency in TLE and JME patients. Fisher exact test was used for statistical analysis. *Inter-IH* inter-ictal headache, *TLE* temporal lobe epilepsy, *JME* juvenile myoclonic epilepsy.

**Figure 5:**
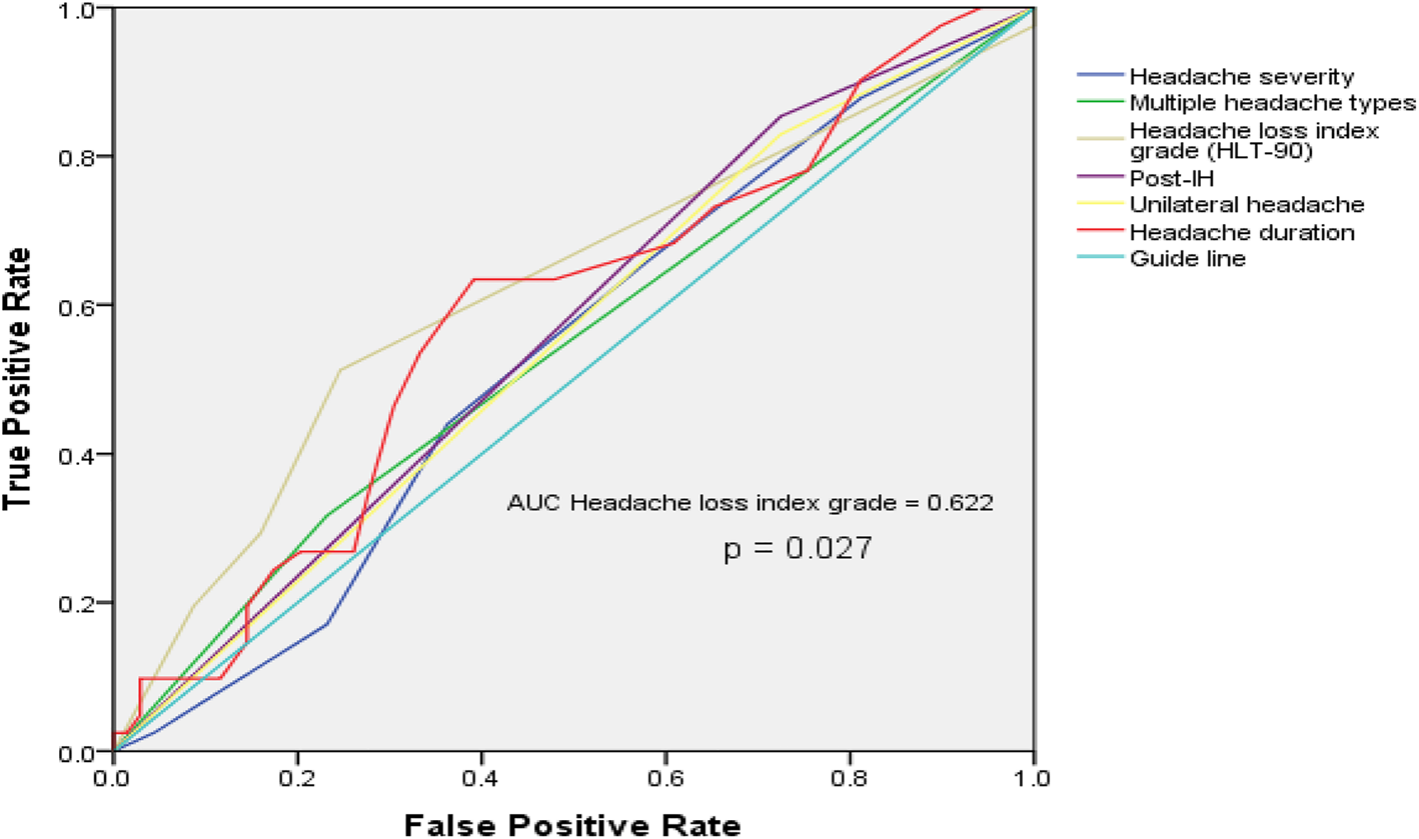
ROC curves of the predictive value of different clinical features of headache on intractable epilepsy in TLE group. For patients presented with more than one type of headache, the most severe type was recorded. *AUC* area under the curve, *ROC* receiver operating characteristic.

We also compared the types of headaches observed in the two groups in our study. Overall, 67 patients (44.37%) had inter-IH, 12 patients (7.95%) had pre-IH, and 118 patients (78.15%) had post-IH in the TLE group; eight patients (26.67%) had inter-IH, 4 patients (13.33%) had pre-IH, and 26 patients (86.67%) had post-IH in the JME group. Of note, 39 and 4 patients were observed with more than one type of headaches in the TLE and JME groups, respectively (Fig. 3). There was no significant difference in terms of family history of headache, headache types, the proportion receiving analgesic therapy, frequency of inter-IH, and QOLIE-10 between TLE and JME patients (Table 1; Fig. 4). TTH were found in 38 (25.17%) TLE patients and 3 (10.00%) JME patients; migraines were found in 10 (6.62%) TLE patients and in 2 (6.67%) JME patients. TLE patients had a higher HLT-90 than JME patients in this study (p = 0.019). In the TLE group, the etiology was infection in 23 patients, and unknown in the remaining patients. The proportion of patients with inter-IH accompanied by nausea in the TLE group was higher than that in the JME group (p = 0.029), and the proportion of patients with headache located in the frontal was lower than that in the JME group (p < 0.05). The other headache features had no statistical difference between the two groups (Table 2). To analyze the predictive value of the headache characteristics on the occurrence of refractory epilepsy, we performed logistic regression analysis. The results suggested that except for the HLT-90 (AUC = 0.622, p = 0.027), other factors were not found to be correlated with the occurrence of refractory epilepsy (Fig. 5).

## Discussion

Headache is one of the most common comorbidities of epilepsy. However, limited studies have reported the incidence of headache in different types of seizures. As a result, we focused on the comparison of the clinical features of headache among TLE and JME patients in this study. TLE is reported to have a higher ratio of refractory epilepsy, and according to the previous literature, intractable epilepsy patients tend to develop more severe headache compared to patients with other types of epilepsies^22^. JME patients are usually therapeutically sensitive to appropriate ASM regimens, and JME and migraine both have genetic susceptibility. Given this, we compared comorbid headache in these two types of epilepsy and further explored the relationship between the clinical features of headache and epilepsy subtypes. Our results demonstrated that the clinical manifestations of headache differed between TLE and JME patients, suggesting that different mechanisms may underline the occurrence of comorbid depending on different types of epilepsy.

Our study showed no significant difference in family history of headache, duration of epilepsy, headache types, proportion receiving analgesic therapy, frequency of inter-IH, and quality of life in QOLIE-10 between TLE and JME patients. However, TLE patients presented an older age, a lower rate of family history of epilepsy, a higher proportion of refractory epilepsy than JME patients. Besides, they had different seizure types and ASM, all due to differences in the epilepsy subtypes. It’s well known that TLE is one of the common types of focal epilepsy and presented with a higher ratio of refractory epilepsy. TLE is usually characterized by recurrent complex partial seizures (with loss of consciousness), and some patients may also have secondary GTCS^23,24^. JME is a type of idiopathic generalized epilepsies characterized by paroxysmal activity of polyspike and slow wave, or spike and wave complexes in EEG and most JME patients respond well to the treatment of an appropriate ASM regimen^25–27^. Some JME patients have a genetic predisposition^28^.

Among all comorbid headaches, migraine has been well characterized. Studies have shown that epilepsy and migraine may share some common pathophysiological processes. Cortical spreading depression (CSD) is considered to be a possible shared pathophysiological mechanism for epilepsy and migraine. Epileptic discharges with or without additional cortical epileptic signs or symptoms might stimulate the onset of CSD, resulting in activation of the trigeminovascular system and headache^29^. In addition, some ASM such as sodium valproate and topiramate anticonvulsants are effective for migraine, suggesting that there is indeed a correlation between the two disorders. In addition to the two drugs mentioned above, ASM with analgesic properties, such as gabapentin may effects the characteristics of the headache. In our study, the proportion of the two groups of patients using the above drugs no difference, so the difference of two groups of patients with headache characteristics could be due to the disease itself.

It was worth noting that the proportion of migraine was lower in the TLE group, and this result was inconsistence with the report that the prevalence of migraine ranged from 9 to 30%^29–33^. It should be noted that the proportion of migraine in our study was obtained in the TLE patients who had headaches, so the real prevalence of migraine should be lower in unselected TLE patients. Of all the migraine patients, only three patients in the TLE group were found with aura. The possible reasons for this are follows: (1) the prevalence of migraine may be different in different epileptic subtypes, as Ito et al. reported that headache characteristics and migraine prevalence were different between TLE and occipital lobe epilepsy^34^. The reason behind the different prevalence of migraine between TLE and JME may involve differences in genetic background, as Schankin et al. inferred that JME patients have strong genetic background, JME appears to be an attractive homogenous subtype of epilepsy for genetic research on migraine. (2) The region of epileptic focus or spreading area of epileptic discharge may have a close relation to the induction of headache^35^. TLE discharges originate in the temporal lobe and may subsequently generalize to the whole brain, and JME is a type of idiopathic generalized epilepsies, the stimulation of the cerebral cortex of seizures may not as strong as in patients with JME, which may account for the lower prevalence of migraine in TLE. Meanwhile, the medial temporal lobe is associated with symptoms such as nausea and vomiting, which may also be the reason why TLE patients have more headaches with emesis symptoms.

The proportion of patients with inter-IH accompanied by nausea was higher and the proportion of patients with headache located in the frontal was lower in the TLE group than in the JME group. The reasons for the difference are not clear. Perhaps it is partly due to that different types of epilepsies had different epileptic foci and abnormal discharge. Our study also showed that patients in the TLE group had a higher HLT-90 than those in the JME group. Since no difference in the frequency of Inter-IH between TLE and JME patients was observed.

Post-IH is the most common comorbid headache of epilepsy. Some hypotheses hold that an increase in blood flow after a seizure or vascular changes triggered by neuronal discharges involving the hypothalamic and brain stem areas may have a role in the induction of Post-IH. A previous study indicated that GTCS and CPS may contribute to the incidence of Post-IH in TLE patients^34^. In our study, patients in the TLE group had more frequent CPS and/or GTCS, while the absence with GTCS episodes was more common in the JME group. Inconsistent with previous reports, the proportion of Post-IH and the characteristics of Post-IH between the two groups had no difference.

We also analyzed the predictive value of the headache characteristics on the occurrence of refractory epilepsy. The results suggested that except for the HLT-90, other factors were not found to be correlated with the occurrence of refractory epilepsy. Further prospective multi-center studies involving larger sample sizes should be conducted to powerfully answer this question.

No ictal headache was observed in our study. Due to the retrospective nature of this study and that most of the patients did not have EEG data at the time of getting headache attack, it was hard to confirm that the patients' headache was ictal headache, which is easy to classify this headache type as Inter-IH. So we may have missed such a difficult diagnosis in our study.

On the basis of our observation that the clinical features of headache differed between TLE and JME patients, we speculated that the nature of headache among epilepsy subtypes may probably not be simple but multifactorial.

Our study has some limitations. Firstly, the limited number of cases in the JME group and the retrospective nature of this study could lead to recall bias. In addition, the patients enrolled in this study were from one of the largest epilepsy centers in China, which had a high proportion of patients with refractory epilepsy, and this could have resulted in the selection bias of patients who received the interview.

## Conclusion

The clinical characteristics of comorbid headache varied in TLE and JME patients. There was no difference in the composition of headache types between TLE and JME patients. TLE patients had a higher ratio of refractory epilepsy, more headache time loss compared with JME patients. HLT-90 was associated with the occurrence of refractory epilepsy in TLE patients. Taken together, we suggested that the comorbid headache may essentially be different between TLE and JME patients.

## Data Availability

The authors declare that the data supporting the findings of this study are available within the article.

## Abbreviations

TLE: Temporal lobe epilepsy
JME: Juvenile myoclonic epilepsy
ICHD- III: International Classification of Headache Disorders-3rd edition
QOLIE-10: Quality Of Life In Epilepsy-10 Inventory
HLT-90: Headache-attributed Lost Time-90 days
Inter-IH: Inter-ictal headache
Peri-IH: Peri-ictal headache
TTH: Tension-type headache
Pre-IH: Pre-ictal headache
Post-IH: Post-ictal headache
GTCS: Generalized tonic–clonic seizures
SD: Standard deviation
CPS: Complex partial seizure
ILAE: International league against epilepsy
ASM: Antiseizure medications
CSD: Cortical spreading depression
OR: Odds ratio
